# Fathers’ perspectives on the diets and physical activity behaviours of their young children

**DOI:** 10.1371/journal.pone.0179210

**Published:** 2017-06-12

**Authors:** Adam D. Walsh, Kylie D. Hesketh, Paige van der Pligt, Adrian J. Cameron, David Crawford, Karen J. Campbell

**Affiliations:** 1 Institute for Physical Activity and Nutrition (IPAN), School of Exercise and Nutrition Sciences, Deakin University, Geelong, Australia; 2 School of Health and Social Development, Deakin University, Geelong, Australia; 3 Global Obesity Centre, Deakin University, Geelong, Australia; Coventry University, UNITED KINGDOM

## Abstract

**Background:**

Children’s learning about food and physical activity is considerable during their formative years, with parental influence pivotal. Research has focused predominantly on maternal influences with little known about the relationships between fathers’ and young children’s dietary and physical activity behaviours. A greater understanding of paternal beliefs regarding young children’s dietary and physical activity behaviours is important to inform the design and delivery of child-focussed health promotion interventions. This study aimed to describe fathers’ perceived roles in their children’s eating and physical activity behaviours. It also sought to document fathers’ views regarding how they could be best supported to promote healthy eating and physical activity behaviours in their young children.

**Methods:**

In depth, semi-structured interviews were conducted with twenty fathers living in socio-economically diverse areas of metropolitan Melbourne, Australia who had at least one child aged five years or less. All interviews were audio recorded, transcribed verbatim and thematically analysed.

**Results:**

Thematic analysis of the transcripts revealed eight broad themes about fathers’ beliefs, perceptions and attitudes towards the dietary and physical activity behaviours of their young children: (i) shared responsibility and consultation; (ii) family meal environment; (iii) parental role modelling; (iv) parental concerns around food; (v) food rewards; (vi) health education; (vii) limiting screen time; and (viii) parental knowledge. Analysis of themes according to paternal education/employment revealed no substantial differences in the views of fathers.

**Conclusions:**

This exploratory study presents the views of a socio-economically diverse group of fathers regarding the dietary and physical activity behaviours of their young children and the insights into the underlying perceptions informing these views. The findings suggest that fathers believe healthy eating behaviours and being physically active are important for their young children. Fathers believe these behaviours can be promoted and supported in different ways including through the provision of appropriate meal and physical activity environments and parental role modelling of desired dietary and physical activity behaviours.

## Introduction

Parents have the largest influence on their children’s food and physical activity behaviours during infancy and early childhood [1, 2]. Children’s learning about food and physical activity is substantial during this time through their observation and imitation of significant others. The influence of parents at this time is considered central [1–5]. The majority of “family” research, however, concentrates on maternal influences or combines the data of both parents, [6–8] precluding an understanding of the independent effects of fathers on young children’s health behaviours. This is likely the result of traditional views of parental roles where mothers have been considered family caretakers and fathers the family breadwinner [9]. The last few decades have witnessed a change in the societal roles of parents, demonstrated by the increasing number of mothers with young children in the workforce [10, 11] resulting in a likely increase in fathers’ participation in family caretaking duties. For example, in a recent study involving over 400 Australian fathers, it was observed that over 50% of fathers reported taking responsibility for some of their young children’s meals [12]. Additionally, much of the literature concentrates on school-aged children, by which time a broad range of other non-family influences may be more important in determining the health behaviours of children [13].

The evidence of the influence of fathers on young children’s health behaviours has been increasing. For example, Wake and colleagues, in their analysis of nationally representative Longitudinal Study of Australian Children data, observed that it was the parenting behaviours and styles of fathers (and not mothers) that were associated with the body mass index (BMI) of their young children [14]. Further, Vollmer and colleagues’ cross-sectional investigation of 150 fathers and their pre-school children observed positive associations between fathers and their children’s vigorous physical activity [15]. Additionally, a recent Australian study of dietary intakes of over 300 fathers and their 20 month-old children, observed positive associations between fathers’ and children’s intake of fruit and sweet snacks independent of mothers dietary intake [16]. Collectively, these studies suggest that fathers’ behaviours may be important influences for their young children’s dietary and physical activity behaviours and subsequent health outcomes. Despite these findings, there is only limited qualitative research on this topic that includes fathers [17]. Qualitative studies are likely to provide an in-depth understanding of the paternal role in the context of young children’s eating and physical activity behaviours and complement the existing evidence from quantitative research by providing preliminary insights into this area and contribute to hypothesis development for future studies.

A recent example of qualitative work investigating young children’s lifestyle behaviours that included fathers is that by Khandpur and colleagues. That study investigated food parenting practices employed by fathers to shape their young children’s dietary behaviours. Twenty food parenting practices were identified, with the most common food parenting practices used including having food rules, letting children dictate food preferences, making healthy foods available, feeding on a schedule, incentivising food consumption and pressuring children to eat.[18]. Additionally, Vollmer and colleagues, in their investigation of fathers’ perceptions about their role and feeding practices, observed that it was fathers’ positive perception of their role at mealtimes (and not their appraisal of maternal perceptions of their role at mealtimes) that was associated with lower use of controlling feeding practices such as pressure to eat or food restriction in their preschool children [19]. While these studies add to the limited literature regarding paternal views about the health behaviours of their young children, they investigated only food parenting practices. Further, they did not consider other potentially important influences on young children’s diets such as fathers’ dietary intakes, nor did they consider fathers’ influence on their young children’s physical activity behaviours. Given this topic is essentially unexplored, providing an understanding of fathers’ views and beliefs about the dietary and physical activity behaviours of their young children will enhance our understanding of the paternal role in this context.

Accordingly, the aims of this qualitative study were to assess fathers’ beliefs and perceived roles in the eating and physical activity behaviours of their young children. This study also aimed to examine fathers’ views regarding the type of support they require to best promote healthy eating and physical activity behaviours in their young children.

## Methods

### Participant sampling and recruitment

Fathers were recruited via 20 childcare centres and kindergartens using flyers distributed through the centre’s usual parental communication method (e.g. in communication pockets or via email). The childcare centres and kindergartens were based in Moreland City Council in the North-Western Department of Education and Early Childhood Development region of greater metropolitan Melbourne, Australia. This demographic area comprises approximately 65 low, 32 medium and 50 high socioeconomic suburbs as defined by the Australian Bureau of Statistics Socio-Economic Indexes for Areas [20]. Eligibility criteria were having at least one child aged five years or less and English literacy. Participant recruitment occurred through a combination of purposive stratified sampling and snowball sampling techniques. Purposive sampling was used to allow a comparatively small number of participants to be recruited who could provide in-depth information across a diversity of demographic backgrounds including education level and occupation [21]. Snowball sampling was employed so that existing study participants could approach possible participants through their existing social networks for recruitment into the study. Sample recruitment was guided by demographic variation and as such two waves of recruitment were employed. The first wave included an equal number of centres from all socioeconomic areas. As the majority of participants recruited in this first wave were from high socioeconomic suburbs, the second wave of recruitment focussed on centres in low socioeconomic suburbs. All fathers who responded to the flyer were screened for eligibility and provided with a plain language statement and consent form via email. All participants provided written, informed consent to participate and have the interview digitally recorded. Consent forms were collected via return email or post prior to the interview being conducted. Participants were reimbursed with a store voucher to the value of twenty dollars in appreciation of their time. Recruitment and data collection were conducted during September 2015 through March 2016. Ethics approval for this study was obtained from the Deakin University Human Research Ethics Committee (approval number HEAG-H 26_2015) and the Victorian Government Department of Education and Training (approval number 2015_002788).

### Data collection and analysis

Data were collected using individual face to face interviews to enable flexibility and greatest opportunity to engage with fathers. Participants nominated a location where they felt most comfortable to undertake the interview (e.g. participants’ workplace or local cafes). Semi-structured interviews (comprising 19 questions) were used to ensure all questions were addressed and to enable comparability of data [22]. Interviews were conducted by a qualified dietitian (ADW) with interview questions exploring attitudes and beliefs around responsibility for decisions in the home environment (for example, w*hat involvement in the decision making about the food/s your family eat do you have*?); role modelling food behaviours (for example, *how important do you think it is to practice what you say or actively role model how you would like your child to behave*?); rules at mealtimes (for example, *what rules*, *if any*, *do you have in your house about your children’s food or eating / meal times*?); contribution towards physical activity and role modelling physical activity behaviours (for example, *how do you spend time with your children when they are active*?) and exploration of the type of support fathers feel they need to best promote healthy behaviours in their young children (for example, *what sort of information or ideas would you be interested in knowing more about with respect to ideas on how to raise healthy*, *active kids*?).The recorded interviews lasted approximately 30–40 minutes and were transcribed verbatim. Participants completed a brief questionnaire including questions about age; highest level of schooling; employment status; marital status; number and ages of children and time as primary caregiver in a typical week (defined as time, in hours, where fathers were the sole caregiver).

Inductive thematic analysis was employed (by ADW) to identify common and unique patterns that extended across the entire set of interviews. Thematic analysis was guided by techniques described by Braun and Clarke [23] whereby codes were generated from the data in an open fashion with no pre-set coding (using NVivo10 software—QSR International). The codes were then reviewed (by ADW) to allow generation of themes and sub-themes until no new themes were identified from the transcripts. Each theme was reviewed for congruency with the transcripts (by ADW) and then peer checked by another member of the research team (PvdP; qualified dietitian; experienced in qualitative research). Any differences in coding and interpretation were resolved by reviewing the interview data again and further discussing coding until agreement was reached. Analysis of the identified themes according to participant socioeconomic position (SEP; low vs high) was undertaken. Participant SEP was defined according to highest level of schooling with high SEP defined as University education and low SEP defined as non-University education. Anonymity of participants was maintained through the use of de-identified data.

## Results

Twenty-three fathers responded to the recruitment flyer with a total of 20 fathers interviewed (three did not schedule a time for an interview, despite repeated contact attempts). Participant characteristics are presented in Table 1. The mean age of fathers was 40 years with the majority (16/20) aged between 32 and 42 years. All fathers were married or living in a de facto relationship, although one father was separated from the biological mother of his child and had shared custody. Nine fathers were university educated, seven held a trade qualification and four attended high school only. Almost all fathers (18/20) were the primary caregiver for their children for some time during a typical week with 10 fathers engaging in primary care for more than 10 hours in a typical week. The emergence of new themes slowed after 14 interviews. Data continued to be collected until no new themes emerged and data saturation was considered to have occurred [24].

**Table 1:** Participant characteristics.

Total sample (n = 20)	
***Fathers’ age (years) (mean*, *SD)***	40.0 (5.1)
***Education level obtained (%)***	
University	45
Trade/apprenticeship	35
High School completed	15
High School incomplete	5
***Employment status (%)***	
Employed / self-employed (full time)	90
Employed / self-employed (part time)	5
Unemployed	5
***Marital Status (%)***	
Married	75
De Facto	25
***Age(years) of target child (mean*, *SD)***	3.6 (1.0)
***Sex of target child (%)***	
Girl	60
***Families with other children (%)***	70
One other child	45
Two other children	20
Three other children	5
***Sex of other children (%)***	
Girl	60
***Age (years) of other children (mean*, *SD)***	
First other child	5.85 (3.0)
Second other child	6.1 (2.1)
Third other child	7 (-)
***Living arrangement with target child (%)***	
Residential, child and wife/partner	95
Non-residential, shared custody	5

Thematic analysis of the transcripts revealed eight broad themes about fathers’ beliefs, perceptions and attitudes towards the dietary and physical activity behaviours of their young children: (i) shared responsibility and consultation; (ii) family meal environment; (iii) parental role modelling; (iv) parental concerns around food; (v) food rewards; (vi) child health education; (vii) limiting screen time; and (viii) parental knowledge. A summary of themes, sub-themes and sample verbatim quotes are presented in Table 2. Analysis of themes according to paternal education/employment revealed no substantial differences in the views of fathers.

**Table 2:** Summary of themes, sub-themes and sample verbatim quotes.

Themes & sub-themes	Sample Quotes
***Theme 1*: *Shared responsibility & consultation***	
Meal preparation/meal planning/shopping	“I do the majority of the cooking but usually my partner and I will discuss…we’ll discuss what we want and then we’ll also tailor around, a little bit around our daughter’s lunch…” (FA04)
	“It’s my partner’s domain for the most part when it comes to deciding what’s for dinner….. I guess I’ll contribute to other things regarding the meal—like reinforcing rules or whatever, so there is some responsibility there.” (FA17)
Physical activity	“Oh yeah equal, 50/50, whatever you want to call it. It’s pretty important. ‘Cause I think she… you know you see… she enjoys being active so we just try and encourage that.” (FA04)
	“I take a bit of the lead on that when it comes to an organised one. But like I said, it’s a shared thing. My wife does more of the fun ones…..I always try to look outside the boxes and think what would he be interested in?” (FA14)
***Theme 2*: *Family meal environment***	
Family meals	“We make sure that it’s family time so that everyone sits at the table together, so it’s a social event. We make sure that we don’t leave the table at dinnertime anyway until everyone’s finished, so introducing some of those basic sort of politeness and things” (FA02)
	“Yeah we always try to make sure we sit down together. I think that’s pretty important. We’ll all sit down and we’ll make sure, yeah. There’s no TV on, there’s nothing like that, sort of just us.” (FA11)
Meal time rules	“There's normally dessert as in pears and yoghurt or something like that and if you don't eat your vegetables and meat—same as what I was brought up with, I guess, unless it's finished on my plate there won't be any further treat.” (FA06)
	“Sometimes…if one of the kids says “I’m done with this can I have some yoghurt please?” then we’ll reinforce the notion of finishing the first part of the meal before the next bit. If we think it’s been a big serve of the main, then we’ll acknowledge that so there’s a context around why we’re allowing the dessert to happen when the main isn’t finished.” (FA17)
***Theme 3*: *Parental role modelling***	
General	“It’s, you know, we’re role models in all aspects of life and I think healthy eating habits and exercise and the whole package is such a critical part. I think there’s a lot geared back to the parents taking responsibility for that because they’re the ones setting the example of that sort of habits/culture. So yeah, no, it’s a huge part of what we practice and preach.” (FA03)
	“I mean I think kids learn by seeing and hearing. We try to model and it doesn’t always work but we are conscious of leading by example for all the kids.” (FA17)
Meal times	“I think it’s very important and that’s why I always make sure that they see me drinking water, for example. I eat with them for breakfast every morning…we eat that together. We nearly always eat the same thing unless my wife’s home a bit later, I might feed the girls first and then we eat later as parents. So on the whole, yeah, we lead by example and eat the same thing.” (FA02)
	”I've lost count of the amount of times I've eaten a dish with vegetables, a balanced meal, because I'm trying to show XX in particular that it’s the right thing to do.” (FA09)
Meal times (continued)	“I think it’s very important and that’s why I always make sure that they see me drinking water, for example. I eat with them for breakfast every morning…we eat that together. We nearly always eat the same thing unless my wife’s home a bit later, I might feed the girls first and then we eat later as parents. So on the whole, yeah, we lead by example and eat the same thing.” (FA02)
Activity	“It gets back to leading by example…. I don't tell a four year old we need to go and do exercise, but I tell them we need to go for a bike ride or to the park (FA06)
	“It’s just a matter of all parents have a responsibility to make sure their kids enjoy sport and see the value in it. You don’t have to breed elite level AFL sportsmen or cyclists or tennis players, whatever else, but as long as the kids enjoy playing something.” (FA10)
***Theme 4*: *Parental concerns around food***	
Convenience	“I guess people have less time so it’s about food that’s easy to prepare which is processed, which is typically like high in calories and low nutritional value.” (FA12)
	“I guess there’s probably been a sort of an instrumental move over time towards more sort of mass produced food, mass prepared food, and I guess there’s probably some growing awareness of the pitfalls of that.” (FA13)
High Energy/Fast Foods	“They hone in on what they like, particularly speedy foods and treat foods and junk food, unfortunately.” (FA05)
High Energy/Fast Foods (continued)	“…we try to make a concerted effort for our kids to not eat a heap of junk food but we still have it…we realise that kids are going to want it…” (FA10)
Food Marketing	“The ads that you see on T.V. of kids going to Maccas and… that’s the visual thing I see through the media.” (FA04)
	“Kids eating everything that's marketed towards what they like and getting them to get their parents to go to restaurants and stuff, Macca's and stuff, like let's get the kids in and they'll tell the parents where they want to go and the parents don't seem to… not have a say but I reckon they're dictated by where the kids want to go instead of the other way around.” (FA08)
***Theme 5*: *Food as a reward***	
Food reinforcement	“I think we all default to that option as parents. We dangle the carrot of obviously a sweet or treat of some kind—as an example, they might get a small ice-cream for eating most of their dinner for the night. To encourage the children to probably eat the right foods first and foremost, we then dangle the incentive carrot that if you do that then you get a reward after the fact.” (FA03)
	“Generally we would not say you cannot have… oh, maybe we would. Maybe we would say… actually, I can think of examples where we have said "You have to finish what's there if you want to have that." (FA09)
Behaviour reinforcement	“We do use food for bribery. It’s more of a bribery thing rather than a reward thing just to keep her still for five minutes while we get ourselves organised.” (FA04)
Behaviour reinforcement (continued)	Yes. I’d say that with, particularly with McDonalds and those sort of treats. . . we just sort of found and decided that it’s better to use it as a treat, things like McDonalds…..yeah it is used as a treat, a bargaining chip or something like that, yeah. The reverse is true too—“OK well we said if this happened you could have some Maccas but it hasn’t so we’re not having it” (FA10)
***Theme 6*: *Child food education***	
	“One thing that we often use is we often refer to food groups for your strength and your sports and other aspects. So all those elements to culminate, you know, encourage them to… this type of food will actually encourage you to be stronger, better at play.” (FA03)
	“It’s not a common thing but when we do it’s generally about trying new things and learning about the foods we like and don’t like. Sometimes it’s from a health perspective but at a kid’s level. Something like dairy is good for strong bones–stuff like that.” (FA18)
***Theme 7*: *Limiting screen time***	
	“You know, when we were growing up it was all about go outside and play and if someone had a ball then play with that. These days there's four of them crowded around a phone instead of going outside and that's what I find challenging.” (FA05)
	“We're not big fans of getting her in front of the TV. We've had friends with kids of a similar age saying to get her to be quiet put Elmo on for half an hour. We're not big on that. I've had the TV on and she takes a semi interest but she's not going to sit on the couch and be glued to it which is a good thing, I think.” (FA08)
***Theme 8*: *Parental knowledge***	
Physical activity	“A playbook of games that parents could default to or use or utilise at home in some capacity would be quite helpful. ‘Cause often we don’t know the games that they play at care or whatever these activities may be, I think would go a long way to changing people’s habits at home.” (FA03)
	“I'm concerned with how much is too much physical activity as a kid. Obviously she's only pretty young at the moment but when she gets older, primary school age, I have no idea if doing a certain amount of exercise a week is detrimental or healthy” (FA08)
Diet	”…And, are there any sort of foods that are a ‘must have’? Or amounts–he eats so variably from day to day and week to week. So what’s normal I guess…” (FA16)
	“I think maybe ideas around what to do when something isn’t right. Like, my child won’t eat such and such so what can I do kind of thing. They change a lot in a short amount of time and there’s so much information out there it’s hard to know what’s right and what isn’t. A definitive guide on what’s what I suppose.” (FA20)

### Shared responsibility and consultation

Most fathers (16/20) acknowledged their shared responsibility with respect to the dietary and physical activity behaviours of their young children. Involvement in grocery shopping, meal planning and meal preparation were identified as key elements where role sharing was the norm. As one father explained:

‘I mean most of our diet choices are fairly sort of joint decisions between my wife and I at the moment….it does depend on where the workload is. But I’d say we share as equally as possible. Yeah certainly overall it’s an equal role.’ (Father 13, high SEP, two children under five)

Whilst fathers were able to identify that their role may be different to that of their partners (e.g. reinforcing meal-time rules), or that they contribute to a greater/lesser degree (e.g. for household grocery shopping), they identified that the overall responsibility was essentially shared equally. Shared responsibility was also identified in the context of physical activity with many fathers recognising the importance of their children being physically active for health as well as enjoyment. For example:

‘We both think activity is important and so it’s really a 50/50 thing I guess. We’ll talk about the structured stuff together and determine if it’s right but other than that we share that responsibility.’ (Father 17, low SEP, one child under five)

### Family meal environment

Almost universally, fathers (19/20) identified the importance of the family meal environment with respect to their children’s eating behaviours, with fathers infrequently mentioning that they occasionally may not be home in time to share the evening family meal. Two important characteristics within this theme were identified. Firstly, fathers believed family meal times were an important part of the family social fabric; not only sharing a meal, but also engaging in other aspects of social etiquette such as family conversations and the formation of family traditions around meals. As one father describes:

‘We try and aim as a family, especially for meal time/dinner time to sit down as a family and share that as not only a time for eating but more so conversing and communicating with the family. So we see food as coupled with that as part of our rules, tradition that we like to sit down as a family.’ (Father 3, high SEP, one child under five)

Meal time rules were the second characteristic in this theme. The setting of boundaries and expectations with respect to behaviour at meal times; the amount of food eaten and the types of foods eaten were commonly raised. Fathers spoke of these rules as having evolved over time in accordance with their young children’s development and their own past experiences being drawn upon to inform the basis of the rules in place. For example:

‘We’ll usually have dessert, maybe fruit or yoghurt or something similar but unless the main is finished there isn’t any further eating… if they don’t eat because they are not hungry then that’s OK. But there’s no eating later on.’ (Father 17, low SEP, one child under five)

### Parental role modelling

Fathers (18/20) identified parental role modelling as important in the context of diet and physical activity. Fathers indicated their belief that parents have a responsibility as the first role models children are exposed to, with the home the first environment this role modelling occurs. It was acknowledged by most fathers (14/20) that the opportunity to set the expected example and influence the behaviours of their children was ever present as their children learn by observation and imitation of those around them. As one father explained:

‘I think it’s extremely important. Your words, your actions, your behaviours have a great impact because I believe the home is the first school that they attend. So what they learn from us is eventually what their characters will be made up of.’ (Father 1, high SEP, one child under five)

Fathers were conscious of setting the example for their children not only in what they say, but also in their actions at meal times. Most fathers (16/20) reflected that, given busy lifestyles, the opportunities to set examples at meal times may not always present themselves. These same fathers believed their young children learned by observation, and that fathers’ role modelling of desired behaviours at meal times was integral to the establishment of these behaviours. For example:

‘I think it's very important, you can tell your kids to eat vegetables but if you're not eating them yourself it's very hard. They think you're not doing it so why should they. I think it's very important.’ (Father 6, low SEP, two children under five)

Similar ideas emerged around parental role modelling of engagement in physical activity. Fathers were cognisant of the different contexts in which physical activity can occur. They acknowledged the importance of contextual structured activities (e.g. swimming lessons), but believed that incidental activity (e.g. walking to local parks) was an opportunity to role model being active generally. For example:

‘I mean basic stuff, like we’d always encourage walking places that we can locally. I guess that also goes with your role modelling stuff and that we would always try and walk somewhere if we can, especially if it’s just a local activity.’ (Father 13 high SEP, two children under five)

Fathers (13/20) also communicated the need for enjoyment when being active, with the implication being that if children found being active fun they would be more likely to engage. It was in this context that fathers discussed their belief their children were more active when they were involved in the activity also. As this father described:

‘I will instigate the rough and tumble….we can get physical where there’s pushing and shoving….so we try to get as involved as much as you can.’ (Father 1, high SEP, one child under five)

### Parental concerns around food

Fathers (16/20) expressed their concerns about food on a variety of related topics including their children’s exposure to high energy, non-core foods (such as sweet or savoury snacks and sugar sweetened beverages), convenience foods and food marketing. Fathers identified the widespread exposure their children had to high energy, non-core foods even when attempting to limit such foods. Fathers recognised that children enjoy high energy foods but the regular exposure to such foods made it difficult to achieve a balanced diet appropriate for young children. As this father pointed out:

‘…yeah, I guess there’s a lot of junk food I guess….or processed food that’s around, that as a parent it’s hard to sort of direct them—I mean to find that balance.’ (Father 7, high SEP, two children under five)

Linked to these concerns, was the topic of food marketing towards young children. Some fathers (6/20) expressed their concern for such marketing due to its omnipresence and the message it conveys to young children that such foods are considered part of an everyday diet. Fathers aired their concerns regarding feeling ill-equipped to deal with the additional parenting burden of such marketing strategies. For example:

‘…well we try to steer the kids while we can now because they’re still pretty young away from commercial TV because it’s just…the advertising is everywhere and it’s hard enough…’ (Father 10, high SEP, one child under five)

Additionally, fathers (8/20) acknowledged convenience foods contributed to ill-informed choices about their family’s eating behaviours. While fathers were mindful of take away foods also being convenient, they were also conscious of the availability of highly processed, mass-produced foods that suggest greater convenience to busy families. However, these fathers did point out that some highly processed foods weren’t any more convenient than the fresh foods they were replacing.

‘Foods that are meant to make life easy. So instead of a piece of fruit, it’s a roll-up. I guess it’s processed food really–anything that we’re told makes life easier around food choices…’ (Father 19, low SEP, two children under five)

### Food as a reward

The use of food as a reward was described by all but one father although there were some notable differences in the approaches fathers took when using this strategy. For example, food rewards were used as an enticement to eat other foods, which was common in the context of children requesting dessert at dinner time. Some fathers (6/20) indicated that it was difficult to achieve a learning environment where the context of when it is appropriate to have dessert could be taught without disparaging other foods and causing the dessert to be the object of desire. In addition, others added they felt ill-equipped to deal with these dinner-time scenarios and deficient in the skills needed to successfully navigate and teach their children in such circumstances. As this father explains:

‘…I suppose I’m looking for her to eat a bit more and maybe might use that bait with the ice-cream or something like that, but I don't like doing it but I’ve got no other tools.’ (Father 15, low SEP, one child under five)

Food was also used as a reward for non-food related behaviour. Fathers (9/20) described using food (commonly high energy, non-core foods) as a reward or bribe in scenarios where they wanted certain behaviours from their children, such as quiet play/activities or assisting with chores. Fathers lamented the consequences of this action in that the foods used for rewarding desired behaviour had become standard snack-time foods and thus had inadvertently been introduced into the everyday diets of their children.

‘…we probably use it as a reward…it's more a treat and …unfortunately where I think we've gone wrong is treats are now just standard snacks rather than a treat for good behaviour.’ (Father 5, high SEP, one child under five)

### Child food education

It was apparent from the responses of several fathers (10/20) that discussions with their children about food were far more frequent than discussions relating to physical activity or play. Topics of discussion varied from food groups (e.g. fruits and vegetables, breads and cereals), how foods contribute to certain parts of the body (e.g. calcium and strong teeth), to healthy versus unhealthy foods. Fathers were mindful of creating a learning environment for their child when talking about food by attempting to provide a context to any information irrespective of what kind of food was being discussed. For example, five of these fathers felt that negative talk about non-core foods, even if these are less desired foods, fails to educate appropriately about the context of when it is appropriate to have these foods.

‘…what we say to the youngest one, that’s a sometimes food, so sort of understand that you don’t want to talk down that it’s so negative and evil because…then the kids will question why they’re even allowed to eat it.’ (Father 10, high SEP, one child under five)

### Limiting screen time

In discussing children’s activities, some fathers (6/20) raised issues around limiting screen time. While fathers only occasionally raised the importance of not having screen time during family meals, it was commonly discussed by fathers in the context of ensuring their children engaged in appropriate levels of activity and minimising sedentary behaviour. For example:

‘I guess a shared underlying value that too much time indoors is not good, like sitting in front of the TV for extensive periods of time is unhealthy.’ (Father 12, high SEP, one child under five)

Limiting the availability of screen use (time of day/total time) was described by fathers although these limits were not without their own challenges. An important challenge for families related to the ubiquity and portability of electronic devices, such that even when their young children are meant to be engaging in non-screen based activity, the appearance of portable electronic devices was not uncommon.

‘You know, when we were growing up it was all about “go outside and play” and if someone had a ball then play with that. These days there's four of them crowded around a phone instead of going outside and that's what I find challenging.’ (Father 5, high SEP, one child under five)

### Parental knowledge

Almost all fathers (19/20) felt they lacked sufficient knowledge to deal with their children’s nutrition and physical activity needs. It was apparent that fathers called upon their own experiences, as well as discussed these needs with their partners, to provide food and activity environments they believe to be appropriate for their young children. However, fathers stated a desire to have greater knowledge of these matters but also articulated frustration about a lack of useable, or, at times conflicting information, regarding children’s nutrition and physical activity. As this father describes:

‘I mean, there’s so much development that occurs in the first few years–how do we know we’re getting it right? It’d be great to have a guide on what to expect at certain ages and what’s OK and what’s too much.’ (Father 16, low SEP, one child under five)

All fathers described wanting to have access to resources on children’s eating and physical activity that were readily available, evidence-based and offered guidance on appropriate foods and physical activities for children across childhood. Whilst many fathers felt a resource of this type could be electronic, a substantial proportion also believed a hardcopy guide would be useful to access on repeated occasions.

In addition to the identified themes presented here, a small number of individual fathers also raised topics relating to food restriction, physical activity skill development and differences in food preparation on weekends. Due to the small number of fathers that raised these topics, they were not presented here.

## Discussion

This study contributes to the limited work examining fathers’ beliefs and perceived roles in the eating and physical activity behaviours of their young children. Additionally, it provides an understanding of paternal views regarding the type of support fathers require to best promote these behaviours. The findings suggest that fathers believe healthy eating and physical activity behaviours are important for their young children; that fathers believe they can promote and support these behaviours in a number of ways; and that their role in such promotion and support is an important one. The results presented here build on existing qualitative work in this area by expanding our understanding of fathers’ perceptions regarding young children’s eating, as well as contributing unique perspectives on the paternal role in their young children’s physical activity behaviours. It is of interest to note that of the eight themes identified, three related to both diet and activity, four related to diet only and one related to physical activity only.

Fathers acknowledged their shared responsibility with mothers in the context of the dietary and physical activity behaviours of their young children. There has been limited work investigating fathers’ perceptions about young children’s dietary behaviours, with the focus generally on paternal feeding practices, (such as pressure to eat and food restriction), in relation to child weight status [12, 19, 25]. However, emerging research has begun to capture the views of fathers on their roles and responsibilities regarding family meals. For example, Mallan and colleagues identified that perceived responsibility for child feeding, along with a more involved attitude toward their role as a father, were positively related to how often fathers ate meals with their children [26]. Fathers in our study described family meals as an important opportunity to develop family social fabric and developmentally appropriate mealtime rules. This is supported by early work by Horodynski and colleagues, who, in their qualitative study of African-American fathers of toddlers, described mealtime patterns of behaviour, such as the establishment of family customs and the development of routines or rules that govern these customs [27]. There is also limited data reporting fathers’ perceptions about their role in young children’s physical activity behaviours. Whilst fathers’ physical activity has been reported as a probable positive correlate of children’s physical activity, [28] as has parental support for physical activity, [29] to our knowledge, fathers’ perceptions about their roles in young children’s physical activity behaviours have not been studied previously. Ferreira and colleagues, in their review of environmental correlates of physical activity, noted that fathers’ physical activity (and not mothers’) was a consistent positive correlate of physical activity in children [28]. Additionally, Cleland and colleagues, in their longitudinal study of the family physical activity environment observed that paternal direct support was positively associated with weekend MVPA in older boys [29]. However, the observations of the Cleland study and the Ferreira review were in children older than five years of age. Our study contributes to the understanding of fathers’ roles in children’s physical activity behaviour through investigation of paternal beliefs that specifically relate to the younger child. Such investigation has provided unique perspectives not previously examined in fathers of children in this age group. In this study, fathers believed they were responsible for role modelling healthy eating behaviours with importance placed on role modelling at the evening meal. This is somewhat supported by work by Campbell and colleagues, who, in their qualitative study exploring Australian parents’ views of children’s food choices, noted that the majority of parents (mothers) in that study believed parental modelling at meals times to be important [30]. However, unlike role modelling desired eating behaviours at specific times, fathers felt opportunities to role model physical activity behaviours were less restrictive with importance placed on enjoyment and participation. Fathers also believed being physically active, (and consequently role modelling physical activity), in these different contexts, to be beneficial to the development of their children’s physical activity behaviours. This notion is supported by a review of correlates of preschool children’s physical activity by Hinkley and colleagues which reported that children with active parents tended to be more active [31].

While fathers were aware of their role in influencing their children’s diet and physical activity, largely through shared responsibility and modelling, they felt there was a lack of information to guide them. Fathers, in the present study, expressed a desire to be better informed about their children’s diet and physical activity needs through access to reliable information. This is in contrast to recent qualitative work by Tanner and colleagues, where mothers described fathers as disinterested in healthy food provision in comparison to mothers [9]. However, as was the case with some fathers in our study, mothers in the Tanner study also described fathers as being unskilled to provide healthy food options.[9] Lamb postulated that fathers’ engagement with their children is determined by four factors, one of which is adequate parenting skills and that possession of these skills results in a higher likelihood of engagement with their children [32]. Yet the fathers in our study, despite identifying a lack of knowledge and skills at times, were already discussing these needs with their partners. This is supported by recent work by Khandpur and colleagues who described the majority of fathers in their qualitative study as sharing parental responsibility for child feeding with their partners, as well as engaging in discourse to resolve any differences that may arise [33]. That these similarities and differences exist in the current literature on fathers’ involvement and perceptions with respect to their young children’s dietary and physical activity needs is not surprising and indicates that significant further study is warranted.

A number of themes were identified relating to parental food concerns. For example, fathers described their belief that foods that are purported to be more convenient were often highly processed, high in energy and low in nutritional benefit. This finding is of interest given that previous studies have reported parental misconceptions regarding the quality of children’s diets. For example, Kourlaba and colleagues, in their large study of Greek mothers and their children below five years of age, observed that over 80% of mothers overestimated the quality of their child’s diet [34]. The fathers in our study, whilst making no comment on the quality of their children’s diets, clearly felt that the convenience and snack foods entering their children’s diets were of poorer nutritional quality than desired.

Fathers were concerned about food marketing towards children, specifically the efforts of such marketing to undermine parental responsibility for food choices through child requests for particular foods. This belief is aligned with recent research that suggests the marketing and advertising of food products to children, via multiple media platforms, are for foods that are of poor nutritional quality [35–37] and influence children’s food preferences and subsequent requests for such foods [38]. That fathers raise these points, and attempt to provide context to their children about these foods, suggests they are not only active participants in the care and feeding of their young children, but also concerned about the impacts of broader social environments on the dietary behaviours of their children.

The use of food as a reward to encourage consumption of another food was commonplace. The use of food as a reward has been well documented previously [30, 39] and so it is not surprising that all but one father indicated using this strategy. Approximately half the fathers expressed concern that using this approach could result in their children liking the reward food more, and the rewarded food less. Early work investigating this notion has produced mixed results, indicating the positive effect that might be expected (increased consumption of the rewarded food) when offering a reward wasn’t observed, although supporting fathers’ concerns, liking for the reward food did increase [40, 41]. Of importance is that the use of food as a reward has been shown to have associations with obesity-promoting eating behaviours in young children [42–44] and increased BMI *z*-scores in primary school aged children [45]. The fathers in this study, whilst not directly linking the use of food as a reward to their children’s weight status, were concerned regarding the possible development of obesity-promoting eating behaviours such as increased exposure and preference for a reward food. Such concern furthers the previously discussed notion of fathers seeking additional guidance to be better informed about their children’s diet and physical activity needs through access to reliable information.

Fathers’ described limitations they imposed on screen time use such as time of day and duration. Whilst there appears to be a lack of established correlates of preschool children’s sedentary behaviours, [46] fathers’ beliefs regarding limiting screen time use appear to be supported by work of Spurrier and colleagues who observed associations between fewer rules about electronic entertainment and higher indoor sedentary time in their study of 280 preschool children’s home environments [42]. Where limitations on screen-based activities did occur in the context of dietary behaviours, this was generally related to the evening meal. Whilst the intention of these actions may be in the context of family customs, they may also provide benefit with respect to dietary intake. For example, Lloyd and colleagues, in their study of maternal and paternal parenting practices in 70 Australian families, observed that screen time use was inversely associated with children’s energy intake from core foods [47]. Thus, fathers’ actions of limiting screen time during family meals may contribute to both the family social fabric as well as healthier dietary intakes for their young children.

It is of interest, that despite including fathers from different SEP categories, analysis of themes according to paternal education/employment revealed no substantial differences in the views of fathers. Previous work undertaken in fathers of young children has indicated that paternal education moderates the relationships between father and child intakes for savoury snacks and take-away foods [48]. However, work investigating the relationship between parental education and children’s physical activity has shown equivocal results. For example, work by Burgi and colleagues, in their investigation of associations between parental education, parental work status and children’s physical activity, observed no differences in child physical activity irrespective of paternal or maternal education or paternal work status [49]. This is supported by work from Kelly and colleagues who, in their investigation of the effect of SEP on the objectively measured physical activity of four to five-year-old children, observed no relationship between low SEP and lower physical activity behaviours[50]. Contrasting these observations is work by Yang and colleagues, who, in their longitudinal investigation of SEP as a predictor of children’s physical activity, observed that nine year-old boys whose fathers were more highly educated participated in more sport than children of lesser educated fathers [51].Although this study contributes to the literature by examining fathers’ perceptions about the diets and physical activity behaviours of their young children, it is not without limitations. Despite including fathers from a range of SEP, the sample may not be representative of all fathers. It is of interest, however, that the themes identified did not differ according to paternal SEP. That fathers were recruited from childcare centres and kindergartens may limit generalisability in that these fathers may be routinely involved in child rearing duties; those who chose to participate may possibly be more interested in nutrition or physical activity and thus only represent a subgroup of fathers. Accordingly, these results may only be generalizable to fathers who have higher levels of involvement in their children’s mealtimes and play. This study only captured fathers’ perspectives and it is possible that mothers’ perspectives with respect to the identified themes may be different. However, the qualitative nature of these data enriches the authentic understanding of fathers’ perspectives in the child food and activity context and contributes to the knowledge base informing the development of evidence-based child health interventions. Such interventions could investigate family dynamics in the context of individual parenting with respect to young children’s eating and physical activity behaviours. Additionally, coupled with previous findings regarding dietary associations between fathers and their young children’s dietary behaviours [16], the contribution of this study to the understanding of fathers’ perspectives on their young children’s eating and physical activity behaviours, may also improve the ability to engage both parents in the delivery of such family-focused interventions.

## Conclusion

This exploratory study presents the views of a socio-economically diverse group of fathers regarding their perceived roles in the dietary and physical activity behaviours of their young children. Additionally, it provides insights into the underlying perceptions that generate these views. This study reveals that fathers view themselves as active participants in their young children’s dietary and physical activity behaviours. The findings suggest that fathers’ believe healthy eating behaviours and being physically active are important for their young children and that these behaviours can be promoted in a number of ways. Future, family focussed investigations, would benefit from including fathers to allow a thorough understanding of their beliefs and influences regarding children’s health behaviours. This, in turn, would improve the ability to deliver effective family-focused child health interventions.

## Supporting information

S1 Table Additional topics raised by individual / small number of fathers. (PDF)

S1 Appendix Survey questionnaire. (PDF)
