# Supplementary material for: Fathers’ perspectives on the diets and physical activity behaviours of their young children
Source: PLoS One. 2017 Jun 12;12(6):e0179210. doi: 10.1371/journal.pone.0179210 (PMC5467895; doi:10.1371/journal.pone.0179210)
Supplement: S1 Table — (PDF) [file pone.0179210.s001.pdf]

Supplementary Table 1. Additional topics raised by individual / small number of fathers

Topic	Sample Quote/s
Topic: Food restriction	"My wife is yeah, on sweet stuff, very strict. She wouldn't offer my daughter chocolate….my wife's much more mindful about sugars generally…" (FA12)
Topic: Physical activity skill development	"Yeah I guess a combination of both. I mean there's some stuff we do together which we should do more, obviously it's not been the season like swimming, so we'll be doing more of that as that comes about and that is a little bit difficult in that she's learning to swim and being taught so it kind of becomes less productive as an exercise I suppose. But I guess once she's got that skill down pat and is in control, then that will change quite a bit. General play around the house obviously is mutual. We do a fair bit of that. But yeah quite looking forward to doing more of those sort of structured things and being able to practice and do all of that sort of stuff. I guess she'll be five so a lot of sports start around then so we'll definitely start stuff by next year, athletic sort of team sport." (FA13)<br><br>"Probably a combination really. I mean there's some stuff we do together – running around the park and that sort of stuff. Maybe some play around the house – we can do a fair bit of that depending on the day. I have to say I'm looking forward to doing more of those structured activities – where I can get a greater enjoyment out of them. There's only so much of kids' activities you can handle you know. I enjoy it, because it's time with the kids, but after a while it can tire." (FA17)
Topic: Weekend food preparation	"On the weekend I might obviously chime in, especially as time of the year has moved to a spring/summer, on the barbecue more often. So we often find that's a bit of a default setting with barbecues and salads and fresh food in that sort of area." (FA03)<br><br>"I tend to prepare the meals on the weekend." (FA05)<br><br>"….so she'd prepare the meals during the week, but I'll do at least one weekend day." (FA17)
