# Supplementary material for: Fathers’ perspectives on the diets and physical activity behaviours of their young children
Source: PLoS One. 2017 Jun 12;12(6):e0179210. doi: 10.1371/journal.pone.0179210 (PMC5467895; doi:10.1371/journal.pone.0179210)
Supplement: S1 Appendix — (PDF) [file pone.0179210.s002.pdf]

Supplementary Appendix 1 - Fathers' Interview Script

- THANKS for participating in the interview today to share your thoughts with me on your child's eating and activity.
- Please keep in mind that there are NO RIGHT OR WRONG answers to these questions. I'm really just interested in what you think.
- Some of these questions might sound really GENERAL, but that's on purpose, I really just want to know what comes to mind for you.
- Some of the questions might also sound a bit REPETITIVE. I want to make sure you have plenty of opportunity to tell me all of your thoughts and ideas. If you've told me everything you think about a topic already, let me know and we can move onto the next group of questions.
- If you DON'T UNDERSTAND a question, just let me know; if there are any questions you want to SKIP that's OK too, just let me know.
- Finally, you can choose to STOP THE INTERVIEW AT ANY TIME if you do not wish to continue with it.
- I will take some notes during our discussion and I will also be RECORDING OUR TALK on a digital voice recorder so that I can remember what you've told me later on. Is this OK with you?
- Are you ready to begin? Let's get started then:

(Start recording) "This is ID_ _"

1. I'm going to start with some questions about children's eating. By this I mean what they eat, how they eat, how much they eat. So in this context, when I say 'kids eating in Australia' what springs to mind?
   → If necessary can prompt: eg: healthy eating, take-away foods, home meals, convenience, etc.

2. What involvement in the decision making about the food/s your family eat do you have?
   → If necessary can prompt: eg: shopping, cooking

3. What rules do you have in your house about your children's food or eating / meal times?
   → If necessary can prompt: eg: types of snacks, eating all of main meal
   → Who generally makes these rules?
   → Who enforces these rules?
   → Is food used as a reward?

4. What involvement do you have in the grocery shopping for your household?
   → If necessary can prompt regarding contribution to shopping list or how often does shopping.

5. What responsibility do you feel you have (even partially) for the decisions made about food at home?
   → If necessary can prompt: eg: through your actions or requests for particular foods

6. How important do you think it is to practice what you say or actively role model how you would like your child to behave?
   How do you do this?
   - Do you eat with your child?
   - How do you talk about food? – eg: do you mention not getting dessert if vegies are unfinished?

   Why do you do this?
   - May prompt with healthy ideas around eating

7. Now I'm going to ask some questions about children's activity. By this I don't just mean organized physical activity (for example swimming lessons), I mean any activity – for example playground play and rough & tumble play. In this context, what are your thoughts on young children's activity?

8. How do you spend time with your children when they are active?
   Eg: What do you do with them?

9. How do you contribute to your child/ren's physical activity?
   → If necessary can prompt with examples

10. What responsibility do you feel you have (even partially) for the decisions made about physical activity at home?
    → If necessary can prompt: eg: through your actions

11. Do you contribute to any home rules around physical activity?
    → If necessary can prompt: eg: sunny day, need to play outside, restriction of sedentary behaviour activities in preference for physical activity?

12. How important do you think it is to practice what you say or actively role model how you would like your child to behave?
    How do you do this?
    - Are you active with your child?
    - How do you talk about activity? – eg: do you mention being fit and healthy?

    Why do you do this?
    - May prompt with healthy ideas around activity

13. What sort of information or ideas would you be interested in knowing more about with respect to ideas on how to raise healthy, active kids?
    → If necessary can prompt - what sort of information would you find useful?
    → Anything else? (Prompt to completion)

14. How do you prefer to find/receive information on topics that interest you?
    eg: online, print articles

15. If you were to be involved in a program where information on children's eating and activity was provided to you, how would you prefer to receive this information?
    → If necessary can prompt – eg: in a group setting?

16. How often do you think you'd realistically like to participate in this sort of program?
    eg: once off vs regular
    eg: weekly vs monthly

17. If we were to design a program and resources providing information to fathers of young children about eating and physical activity how would you suggest we do that?
    → Anything else? (Prompt to completion)

18. Is there anything else you'd like to add to what we've talked about?
    → Anything else? (Prompt to completion

19. Would you be happy to be contacted again for a follow-up study on the same topic?

THANK YOU SO MUCH FOR YOUR TIME
